# An Interval-Valued Intuitionistic Fuzzy TOPSIS Method Based on an Improved Score Function

**DOI:** 10.1155/2013/879089

**Published:** 2013-12-25

**Authors:** Zhi-yong Bai

**Affiliations:** College of Arts and Design, Beijing Forestry University, 35 Tsinghua East Road, Beijing 100083, China

## Abstract

This paper proposes an improved score function for the effective ranking order of interval-valued intuitionistic fuzzy sets (IVIFSs) and an interval-valued intuitionistic fuzzy TOPSIS method based on the score function to solve multicriteria decision-making problems in which all the preference information provided by decision-makers is expressed as interval-valued intuitionistic fuzzy decision matrices where each of the elements is characterized by IVIFS value and the information about criterion weights is known. We apply the proposed score function to calculate the separation measures of each alternative from the positive and negative ideal solutions to determine the relative closeness coefficients. According to the values of the closeness coefficients, the alternatives can be ranked and the most desirable one(s) can be selected in the decision-making process. Finally, two illustrative examples for multicriteria fuzzy decision-making problems of alternatives are used as a demonstration of the applications and the effectiveness of the proposed decision-making method.

## 1. Introduction

Decision-making is the procedure to find the best alternative among a set of feasible alternatives. TOPSIS, developed by Hwang and Yoon [1], is a well-known multicriteria decision-making method. The basic concept of the TOPSIS method is that the chosen alternative should have the shortest distance from the positive ideal solution and the farthest distance from the negative ideal solution. TOPSIS assumes that each criterion takes either monotonically increasing or monotonically decreasing utility. Therefore, many researchers have extended the TOPSIS approach to fuzzy environment as a natural generalization of TOPSIS models. It has been wildly applied in fuzzy multicriteria decision-making problems. Triantaphyllou and Lin [2] develop a fuzzy version of the TOPSIS approach based on fuzzy arithmetic operations, resulting in a fuzzy relative closeness for each alternative. Chen [3] extends the TOPSIS approach to fuzzy group decision making situations by defining a crisp Euclidean distance between any two fuzzy numbers. Tsaur et al. [4] convert a fuzzy multicriteria decision-making problem into a crisp one by means of centroid defuzzification and then solve the nonfuzzy multicriteria decision-making problem using the TOPSIS approach. In recent years, many researchers have paid great attention to interval-valued fuzzy sets (IVFSs) [5, 6], intuitionistic fuzzy sets (IFSs)/vague sets (VSs) [7, 8] and interval-valued intuitionistic fuzzy sets (IVIFSs) [9], which are all the generalization of the fuzzy set proposed by Zadeh [10], and applied them in decision-making problems. For example, Ashtiani et al. [11] proposed an interval-valued fuzzy technique for order preference by similarity to an ideal solution (TOPSIS) for solving multicriteria decision-making problems. Then, Chen [3] introduced SAW-based and TOPSIS-based multicriteria decision-making methods with score functions and weight constraints and conducted a comparative study through computational experiments under an interval-valued fuzzy framework. Li [12] proposed multiattribute decision-making models and methods using IFSs, and then Li [13] extended the generalized-ordered, weighted, averaging operators to investigate multiattribute decision-making problems using the score function and the accuracy function to rank the IFSs. On the other hand, Ye [14] proposed an improved algorithm for score functions by taking into account the effect of an unknown degree (hesitancy degree) of VSs and a multicriteria decision-making method based on the score function of VSs. Then, Ye [15] presented a multicriteria decision-making method using an improved accuracy function of VSs. Chen [16] established flexible algorithms with SAW and TOPSIS methods by considering both objective and subjective information to compute optimal multicriteria decisions. Chen [17] gave a comparative analysis of score functions for multicriteria decision-making in intuitionistic fuzzy settings. In the context of interval-valued intuitionistic fuzzy sets, Li [18] constructed a pair of nonlinear fractional programming models to calculate the relative closeness coefficient intervals of alternatives to the ideal solutions. In a similar manner, Li [19] developed TOPSIS-based nonlinear-programming methodology. Park et al. [20] extended the TOPSIS method to solve multiple attribute group decision making problems under interval-valued intuitionistic fuzzy environment in which all the preference information provided by the decision-makers is presented as interval-valued intuitionistic fuzzy decision matrices where each of the elements is characterized by an interval-valued intuitionistic fuzzy number (IVIFN), and the information about attribute weights is partially known. Lai and Chen [21] extended a similarity measure in the technique for order preference based on similarity to the ideal solution (TOPSIS) approach by measuring the similarity of each alternative to positive and negative ideal interval-valued fuzzy numbers (IVFNs) and applied the similarity measure between IVFNs to the decision-making process to increase the ability of the process to account for risks in a variable, complex, and uncertain environment.

Consider, that the socioeconomic environment becomes more complex, the preference information provided by decision-makers is usually imprecise; that is, there may be hesitation or uncertainty about preferences because a decision should be made under time pressure and lack of knowledge or data, or the decision-makers have limited attention and information processing capacities. In such cases, it is suitable and convenient to express the decision-makers' preferences by IVIFSs. The fundamental characteristic of the IVIFS is that the values of its membership function and nonmembership function are intervals rather than exact numbers. In order to make comparisons between two IVIFSs, some metric methods were introduced by score functions and accuracy functions [15, 22, 23] and were applied to multicriteria decision-making problems. However, our survey shows that these functions are the vital shortcoming in some cases. Therefore, in this paper we proposed an improved score function and develop an interval-valued intuitionistic fuzzy TOPSIS method based on the improved score function to solve multicriteria decision-making problems in which the performance rating values are expressed by IVIFSs.

The remaining of this paper is organized as follows. In Section 2, we briefly introduce IVIFS and its score functions and accuracy functions.  Section 3 proposes an improved score function of IVIFSs and makes comparisons of score functions and accuracy functions of IVIFSs by an example to illustrate the effectiveness of the proposed score function.  Section 4 develops a TOPSIS method based on the improved score function to solve interval-valued intuitionistic fuzzy multicriteria decision-making problems.  Section 5 investigates two illustrative examples for demonstrating the applications and effectiveness of the proposed decision-making method. The paper is concluded in Section 6.

## 2. IVIFS and Its Score Functions and Accuracy Functions

This section introduces the basic definitions and some score functions and accuracy functions relating to IVIFS, which will be needed in the analysis of the following sections.

**Definition 1 (see [9])**

Let *D*[0, 1] be the set of all closed subintervals of the interval [0, 1] and let *X*(≠*∅*) be a given set. An IVIFS *A* in *X* is defined as

(1) A={〈x,μA(x),vA(x)〉 ∣ x∈X},

where *μ*_*A*_ : *X* → *D*[0,1], *v*_*A*_ : *X* → *D*[0,1] with the condition 0 ≤ sup⁡(*μ*_*A*_(*x*)) + sup⁡(*v*_*A*_(*x*)) ≤ 1 for any *x* ∈ *X*.

The intervals *μ*_*A*_(*x*) and *v*_*A*_(*x*) denote, respectively, the membership degree and the nonmembership degree of the element *x* to the set *A*. Thus, for each *x* ∈ *X*, *μ*_*A*_(*x*) and *v*_*A*_(*x*) are closed intervals and their lower and upper end points are, respectively, denoted by *μ*_*AL*_(*x*), *μ*_*AU*_(*x*), *v*_*AL*_(*x*), and *v*_*AU*_(*x*). We can denote

(2) A={〈x,[μAL(x),μAU(x)],[vAL(x),vAU(x)]〉 ∣ x∈X},

where 0 ≤ *μ*_*AU*_(*x*) + *v*_*AU*_(*x*) ≤ 1, *μ*_*AL*_(*x*) ≥ 0, *v*_*AL*_(*x*) ≥ 0.

For each element *x*, we can compute the unknown degree (hesitancy degree) of an intuitionistic fuzzy interval of *x* ∈ *X* in *A* defined as follows:

(3) πA(x)=1−μA(x)−vA(x)=[1−μAU(x)−vAU(x),1−μAL(x)−vAL(x)].

Especially, if *μ*_*A*_(*x*) = *μ*_*AL*_(*x*) = *μ*_*AU*_(*x*) and *v*_*A*_(*x*) = *v*_*AL*_(*x*) = *v*_*AU*_(*x*), then the given IVIFS *A* is reduced to an ordinary IFS.

For two IVIFSs *A* = {〈*x*, [*μ*_*AL*_(*x*), *μ*_*AU*_(*x*)], [*v*_*AL*_(*x*), *v*_*AU*_(*x*)]〉 | *x* ∈ *X*} and *B* = {〈*x*, [*μ*_*BL*_(*x*), *μ*_*BU*_(*x*)], [*v*_*BL*_(*x*), *v*_*BU*_(*x*)]〉 | *x* ∈ *X*}, the following two relations are defined [9]:

- *A*⊆*B* if and only if *μ*_*AU*_(*x*) ≤ *μ*_*BU*_(*x*), *μ*_*AL*_(*x*) ≤ *μ*_*BL*_(*x*), *v*_*AU*_(*x*) ≥ *v*_*BU*_(*x*), and *v*_*AL*_(*x*) ≥ *v*_*BL*_(*x*) for any *x* ∈ *X*;
- *A* = *B* if and only if *μ*_*AU*_(*x*) = *μ*_*BU*_(*x*), *μ*_*AL*_(*x*) = *μ*_*BL*_(*x*), *v*_*AU*_(*x*) = *v*_*BU*_(*x*), and *v*_*AL*_(*x*) ≥ *v*_*BL*_(*x*) for any *x* ∈ *X*.

An IVIFS value is denoted by *A* = ([*a*, *b*], [*c*, *d*]) for convenience. In order to make comparisons between two IVIFSs, some metric methods should be introduced by the following score function and accuracy functions.

(1) Score function [23]:
  (4) S(A)=a−b+c−d2, S(A)∈[−1,+1].
(2) Accuracy function [23]:
  (5) H(A)=a+b+c+d2, H(A)∈[0,1].
(3) Novel accuracy function [15]:
  (6) M(A)=a−(1−a−c)+b−(1−b−d)2,             M(A)∈[−1,+1].
(4) Another accuracy function [22]:
  (7) L(A)=a+b−d(1−b)−c(1−a)2, L(A)∈[−1,+1].

From the above function forms of metric methods, we can see that the functions (1)–(3) fail to rank correctly when the sum of lower bound and upper bound of membership and nonmembership degrees is equal, respectively. In the function (4), the unknown degree has not been considered sufficiently, so indeterminacy information has not been extracted completely.

## 3. Improved Score Function

Let *A* = ([*a*, *b*], [*c*, *d*]) be an IVIFS value, its improved score function based on the unknown degree is proposed by the following formula:

(8) I(A)=a+a(1−a−c)+b+b(1−b−d)2,

where *I*(*A*)∈[0, 1]. Especially, when *a* = *b* and *c* = *d*, an IVIFS is degenerated to an IFS, and then we can find that the improved score function of IVIFS is degenerated to the score function of IFS [24].

To illustrate the effectiveness of the proposed score function, let us consider the following example.

**Example 2**

If interval-valued intuitionistic fuzzy values for two alternatives are *A* = ([0.3, 0.7], [0.1, 0.3]) and *B* = ([0.4, 0.6], [0.0, 0.4]), the desirable alternative is selected in accordance with score functions.

For the comparisons of various metric methods, we can calculate the function values of various methods as shown in Table 1.

From Table 1, the proposed score function can rank correctly from the relationship between *A* and *B*. Therefore, the alternative *B* is better than the alternative *A*. But other functions cannot rank correctly; thus, we do not know which alternative is better. From the point of view of intuition, the relationship between *A* and *B* demonstrates that the proposed score function is reasonable.

## 4. TOPSIS Method Based on the Improved Score Function

TOPSIS method, a compromising model developed by Gorzałczany [5], is widely used in multicriteria decision-making problems. In this section, we develop a TOPSIS method to solve multicriteria decision-making problems in which all preference information provided by decision-makers is expressed as interval-valued intuitionistic fuzzy decision matrices where each of the elements is characterized by IVIFS value, and the information about criterion weights is known. We apply the proposed score function to calculate the separation measures of each alternative from the positive and negative ideal solutions to determine the relative closeness coefficients.

In a multicriteria decision-making problem, suppose that there exists a set of alternatives *A* = {*A*_1_, *A*_2_, …, *A*_*m*_}. Each alternative is assessed on *n* criteria, which are denoted by *C* = {*C*_1_, *C*_2_, …, *C*_*n*_}. The characteristics of an alternative *A*_*i*_ with respect to a criterion *C*_*j*_ can be represented by an IVIFS value *x*_*ij*_ = ([*a*_*ij*_, *b*_*ij*_], [*c*_*ij*_, *d*_*ij*_]) (*i* = 1, 2,…, *m*; *j* = 1,2,…, *n*), which can represent the membership degree and nonmembership degree of the alternative *A*_*i*_ ∈ *A* with respect to the criterion *C*_*j*_ ∈ *C* for the fuzzy concept “excellence.” The interval-valued intuitionistic fuzzy decision matrix *D*_*m*×*n*_(*x*_*ij*_) is defined as the following form:

(9) Dm×n(xij)=[([a11,b11],[c11,d11])([a12,b12],[c12,d12])⋯([a1n,b1n],[c1n,d1n])([a21,b21],[c21,d21])([a22,b22],[c22,d22])⋯([a2n,b2n],[c2n,d2n])⋮⋮⋱⋮([am1,bm1],[cm1,dm1])([am2,bm2],[cm2,dm2])⋯([amn,bmn],[cmn,dmn])].

Based on the improved score function, we convert the interval-valued intuitionistic fuzzy decision matrix *D*_*m*×*n*_(*x*_*ij*_) into the following score matrix *R*_*m*×*n*_(*I*_*ij*_(*x*_*ij*_)):

(10) Rm×n(Iij(xij))  =[I11(x11)I12(x12)⋯I1n(x1n)I21(x21)I22(x22)⋯I2n(x2n)⋮⋮⋱⋮Im1(xm1)Im2(xm2)⋯Imn(xmn)].

Assume that the weight of the criterion *C*_*j*_  (*j* = 1, 2, …, *n*), entered by the decision-maker, is *w*_*j*_, *w*_*j*_∈[0, 1], and ∑_*j*=1_^*n*^ *x*_*j*_ = 1.

Then, the positive ideal solution for the alternatives is denoted by *A* ^+^ = {〈*C*_*j*_, [1,1], [0,0]〉 | *C*_*j*_ ∈ *C*}, *j* = 1, 2,…, *n*, and the negative ideal solution for the alternatives is denoted by *A* ^−^ = {〈*C*_*j*_, [0,0], [1,1]〉 | *C*_*j*_ ∈ *C*} and *j* = 1,2,…, *n*.

Thus, the score function-based separation measures *d*_*i*_^+^(*A* ^+^, *A*_*i*_)  and  *d*_*i*_^−^(*A* ^−^, *A*_*i*_) of each alternative from the positive ideal and negative ideal solutions, respectively, are derived by the following forms:

(4) di+(A+,Ai)=∑j=1n[wj(1−Iij(xij))]2,di−(A−,Ai)=∑j=1n(wjIij(xij))2.

Hence, the relative closeness of an alternative *A*_*i*_ with respect to the positive ideal solution *A* ^+^ is defined as the following general formula:

(12) Ci(Ai)=di−(A−,Ai)di+(A+,Ai)+di−(A−,Ai),

where *C*_*i*_(*A*_*i*_)  (*i* = 1, 2, …, *m*) is the relative closeness coefficient of *A*_*i*_ with respect to the positive ideal solution *A* ^+^ and 0 ≤ *C*_*i*_(*A*_*i*_) ≤ 1. Therefore, the alternatives can be ranked according to the descending order of *C*_*i*_(*A*_*i*_). Moreover, the alternative with the highest value of *C*_*i*_(*A*_*i*_) will be the best choice.

## 5. Illustrative Examples

In this section, two examples for multicriteria fuzzy decision-making problems of alternatives are used as a demonstration of the applications and the effectiveness of the proposed decision-making method.

### 5.1. Example 1

Let us consider the decision-making problem discussed in [15] and make a new example for computer animation competition. There is an animation expert, who wants to select a best computer animation work for the further reward. There is a computer animation competition with four possible alternatives *A*_*i*_ (*i* = 1,2, 3,4). The expert has to make a decision according to the following three criteria: (1)  *C*_1_ is the artistic appeal; (2)  *C*_2_ is the visual effect; and (3)  *C*_3_ is the creative script. The criterion weight is given by *W* = (0.35,0.25,0.40). The alternative 4 *A*_*i*_ (*i* = 1,2, 3,4) is to be evaluated using the interval-valued intuitionistic fuzzy value by the decision-maker under the above three criteria, as listed in the following decision matrix *D*_4×3_(*x*_*ij*_):

(13) D4×3(xij)=[([0.4,0.5],[0.3,0.4])([0.4,0.6],[0.2,0.4])([0.1,0.3],[0.5,0.6])([0.6,0.7],[0.2,0.3])([0.6,0.7],[0.2,0.3])([0.4,0.7],[0.1,0.2])([0.3,0.6],[0.3,0.4])([0.5,0.6],[0.3,0.4])([0.5,0.6],[0.1,0.3])([0.7,0.8],[0.1,0.2])([0.6,0.7],[0.1,0.3])([0.3,0.4],[0.1,0.2])].

Then, we utilize the developed approach to obtain the most desirable alternative(s).

By using (8), we convert the interval-valued intuitionistic fuzzy decision matrix *D*_4×3_(*x*_*ij*_) into the following score matrix *R*_4×3_(*I*_*ij*_(*x*_*ij*_)):

(14) R4×3(Iij(xij))=[0.53500.58000.23500.71000.71000.68500.51000.60000.68000.82000.74000.5200].

By using (4), we can compute *d*_*i*_^+^(*A* ^+^, *A*_*i*_) and *d*_*i*_^−^(*A* ^−^, *A*_*i*_)  (*i* = 1, 2, 3, 4) as follows: *d*_1_^+^(*A* ^+^, *A*_1_) = 0.3621, *d*_2_^+^(*A* ^+^, *A*_2_) = 0.1773, *d*_3_^+^(*A* ^+^, *A*_3_) = 0.2362, and *d*_4_^+^(*A* ^+^, *A*_4_) = 0.2123; *d*_1_^−^(*A* ^−^, *A*_1_) = 0.2548, *d*_2_^−^(*A* ^−^, *A*_2_) = 0.4103, *d*_3_^−^(*A* ^−^, *A*_3_) = 0.3583, and *d*_4_^−^(*A* ^−^, *A*_4_) = 0.3998.

By applying (12), we have the following closeness coefficient *C*_*i*_(*A*_*i*_) (*i* = 1,2, 3,4):  *C*_1_(*A*_1_) = 0.4130, *C*_2_(*A*_2_) = 0.6983, *C*_3_(*A*_3_) = 0.6026, and *C*_4_(*A*_4_) = 0.6532.

Therefore, the ranking order of the four alternatives is *A*_2_, *A*_4_, *A*_3_, and *A*_1_; obviously, amongst them, *A*_2_ is the best alternative. These results are in agreement with the ones obtained in [15], similarly.

### 5.2. Example 2

We consider the same problem as in [23] and make another example. The alternative *A*_*i*_ (*i* = 1,2,…, 5), the appropriate criterion *C*_*j*_(*j* = 1, 2,…, 6), and the criterion weight *W* = (0.20, 0.10, 0.25, 0.10, 0.15, 0.20) are given for the decision-maker, and then, the decision matrix *D*_5×6_(*x*_*ij*_) is constructed by using interval-valued intuitionistic fuzzy values tabulated as Table 2. The decision-maker has to perform the decision process and select the best alternative from these alternatives according to the given criteria.

Then, the proposed method is applied to solve this problem.

By using (8), we convert the interval-valued intuitionistic fuzzy decision matrix *D*_5×6_(*x*_*ij*_) into the following score matrix *R*_5×6_(*I*_*ij*_(*x*_*ij*_)):

(15) R5×6(Iij(xij))  =[0.32000.71000.55500.82000.23500.67500.71000.68000.71000.77500.38000.68500.53500.82000.60000.74000.53500.54000.71000.70000.82000.52000.68000.82000.57000.43000.74000.79000.71000.6250].

By using (4), we can compute *d*_*i*_^+^(*A* ^+^, *A*_*i*_) and *d*_*i*_^−^(*A* ^−^, *A*_*i*_)  (*i* = 1, 2, 3, 4) as follows: *d*_1_^+^(*A* ^+^, *A*_1_) = 0.2223, *d*_2_^+^(*A* ^+^, *A*_2_) = 0.1509, and *d*_3_^+^(*A* ^+^, *A*_3_) = 0.1816, *d*_4_^+^(*A* ^+^, *A*_4_) = 0.1104, *d*_5_^+^(*A* ^+^, *A*_5_) = 0.1511; *d*_1_^−^(*A* ^−^, *A*_1_) = 0.2336, *d*_2_^−^(*A* ^−^, *A*_2_) = 0.2904, and *d*_3_^−^(*A* ^−^, *A*_3_) = 0.2535, *d*_4_^−^(*A* ^−^, *A*_4_) = 0.3273, *d*_5_^−^(*A* ^−^, *A*_5_) = 0.2868.

Then, by applying (12), we obtain the following closeness coefficient *C*_*i*_(*A*_*i*_) (*i* = 1,2, 3,4, 5): *C*_1_(*A*_1_) = 0.5124, *C*_2_(*A*_2_) = 0.  6580, *C*_3_(*A*_3_) = 0.5826, *C*_4_(*A*_4_) = 0.7477, and *C*_5_(*A*_5_) = 0.6550.

Therefore, the ranking order of the five alternatives is *A*_4_, *A*_2_, *A*_5_, *A*_3_, and *A*_1_; obviously, amongst them, *A*_4_ is the best alternative. These results are in agreement with the ones obtained in [23].

## 6. Conclusion

In this paper, we proposed an improved score function of IVIFS and an interval-valued intuitionistic fuzzy TOPSIS method based on the proposed score function. In the proposed TOPSIS method, we apply the proposed score function to calculate the separation measures of each alternative from the positive and negative ideal solutions to determine the relative closeness coefficients. According to the values of the closeness coefficients, the alternatives can be ranked and the most desirable one(s) can be selected in the decision-making process. Finally, two illustrative examples illustrated the applications and efficiency of the developed approach. In the future, we will continue working on the application of the proposed method to other domains.

## Figures and Tables

**Table 1:** Function values of various methods.

Methods	Function values
*S*(·)	*S*(*A*) = *S*(*B*) = 0.3
*H*(·)	*H*(*A*) = *H*(*B*) = 0.7
*M*(·)	*M*(*A*) = *M*(*B*) = 0.2
*L*(·)	*L*(*A*) = *L*(*B*) = 0.42
*I*(·)	*I*(*A*) = 0.59, *I*(*B*) = 0.62

**Table 2:** Decision matrix *D*_5×6_ (*x*_*ij*_).

	*C*_ 1_	*C*_ 2_	*C*_ 3_	*C*_ 4_	*C*_ 5_	*C*_ 6_
*A*_ 1_	([0.2, 0.3], [0.4, 0.5])	([0.6, 0.7], [0.2, 0.3])	([0.4, 0.5], [0.2, 0.4])	([0.7, 0.8], [0.1, 0.2])	([0.1, 0.3], [0.5, 0.6])	([0.5, 0.7], [0.2, 0.3])
*A*_ 2_	([0.6, 0.7], [0.2, 0.3])	([0.5, 0.6], [0.1, 0.3])	([0.6, 0.7], [0.2, 0.3])	([0.6, 0.7], [0.1, 0.2])	([0.3, 0.4], [0.5, 0.6])	([0.4, 0.7], [0.1, 0.2])
*A*_ 3_	([0.4, 0.5], [0.3, 0.4])	([0.7, 0.8], [0.1, 0.2])	([0.5, 0.6], [0.3, 0.4])	([0.6, 0.7], [0.1, 0.3])	([0.4, 0.5], [0.3, 0.4])	([0.3, 0.5], [0.1, 0.3])
*A*_ 4_	([0.6, 0.7], [0.2, 0.3])	([0.5, 0.7], [0.1, 0.3])	([0.7, 0.8], [0.1, 0.2])	([0.3, 0.4], [0.1, 0.2])	([0.5, 0.6], [0.1, 0.3])	([0.7, 0.8], [0.1, 0.2])
*A*_ 5_	([0.5, 0.6], [0.3, 0.5])	([0.3, 0.4], [0.3, 0.5])	([0.6, 0.7], [0.1, 0.3])	([0.6, 0.8], [0.1, 0.2])	([0.6, 0.7], [0.2, 0.3])	([0.5, 0.6],[0.2, 0.4])
